# Dental Caries Prevalence and Experience (ICDAS II Criteria) of 5-, 12- and 15-Year-Old Children and Adolescents with an Immigrant Background in Greece, Compared with the Host Population: A Cross-Sectional Study

**DOI:** 10.3390/ijerph19010014

**Published:** 2021-12-21

**Authors:** Iliana Diamanti, Elias D. Berdouses, Katerina Kavvadia, Konstantinos N. Arapostathis, Argy Polychronopoulou, Constantine J. Oulis

**Affiliations:** 1 Department of Preventive and Community Dentistry, School of Dentistry, National and Kapodistrian University of Athens, 11527 Athens, Greece; argypoly@dent.uoa.gr; 2 Independent Researcher, 15231 Athens, Greece; elias@paedoclinic.gr; 3 Department of Dentistry, European University Cyprus, 6 Diogenous Str., 2404 Nicosia, Cyprus; k.kavvadia@euc.ac.cy; 4 Department of Paediatric Dentistry, School of Dentistry, Aristotle University of Thessaloniki, 54124 Thessaloniki, Greece; koarap@dent.auth.gr; 5 Department of Paediatric Dentistry, School of Dentistry, National and Kapodistrian University of Athens, 11527 Athens, Greece; cjoulis@paedoclinic.gr

**Keywords:** dental caries, ICDAS-II criteria, immigrants, children, adolescents, comparative study, risk indicators, ethnic background

## Abstract

Evidence regarding disparities in oral health among native and immigrant child and adolescent populations in Europe is scarce. The present study aimed to determine the caries status of 5-, 12-, and 15-year-olds with an immigrant background in Greece in relation to their sociodemographic characteristics and compare their caries levels with those of their native Greek peers. A sample of 707 immigrants underwent clinical examination for caries (ICDAS II criteria), followed by a calculation of caries prevalence and experience estimates (2013–2014). Multivariable regression models assessed the effect of ethnic background on the caries experience (d_3–6_mfs/D_3–6_MFS) of the total (707 immigrants and 3702 Greeks) population, and the association between parental education level and the immigrants’ d_3–6_mfs/D_3–6_MFS. Among the 5- and 12-year-olds, those with an immigrant background demonstrated higher caries rates and had unfulfilled treatment needs at higher proportions. Among 15-year-old adolescents, no considerable differences in caries status were observed according to ethnic background (native Greek or immigrant). The strength of the association between immigrant background and caries experience levels attenuated gradually with increasing age (IRR = 1.61, 1.27 and 1.10, and *p* = 0.001, *p* = 0.006 and *p* = 0.331 for 5-, 12- and 15-year-olds, respectively). Among the immigrants, adolescents with less educated mothers exhibited elevated caries levels. Public health strategies should prioritize immigrant children and early adolescents in order to reduce the existing ethnic disparities in oral health.

## 1. Introduction 

During the past three decades, Greece has been transformed from a migrant-sending country to a migrant-receiving country. Migrant inflows accelerated with the collapse of socialist regimes in Central and Eastern Europe at the beginning of the 1990s, with Albania predominating as a source country [1]. According to the most recent census (2011), 7.8% (862,361 individuals) are non-Greek citizen residents, with Albanians continuing to dominate the ‘settled’ migrant population. Specifically, they comprised 52.7% of the foreign population, distantly followed by Bulgarians and Romanians, who accounted for 8.3% and 5.1%, respectively [1]. Considering the size of the child immigrant population, it was estimated that during the years 2010–2011, 11.2% (154,120 individuals) of the students attending pre-primary to upper secondary education levels in the public school sector were foreign in origin [2], with the majority constituting children with an Albanian background [3].

Most theoretical constructs of immigrant health rely on ‘acculturation’, the process of modifying cultural standards when coming in direct contact with a new culture, within the broader social environment of the host country. These cultural transitions can either be favourable or harmful to oral health status [4]. Globally, highly accultured migrant populations were observed to maintain better oral health and adopt more favourable oral health practices [4]. In Europe, documentation about the oral health level of populations with an immigrant background is scarce and sporadic. Evidence derived from studies conducted within Western European countries consistently suggested that caries prevalence is higher among immigrant children and early adolescents younger than 12 years of age compared with the respective native inhabitants [5]. Nevertheless, the evidence is less explicit for older immigrant adolescents, as discrepancies were observed regarding caries experience levels across different European countries. Some studies demonstrated dental health among certain immigrant sub-groups approximating or even being superior to that of native residents, in contrast to other reports in which adolescents with an immigrant background presented with an inferior dental status to their host peers [5].

Caries level estimates rely strongly on the protocol of examination and the detection criteria applied [6]. In recent years, a declining prevalence of caries at the cavitation stage in several childhood populations, with a concomitant raise in the number of incipient caries lesions [7], has been observed, leading to the establishment of the ICDAS criteria for the detection and classification of caries lesions, which include the recording of early caries lesions [8]. Recording enamel caries in epidemiological studies enhances their sensitivity, particularly in populations where the initial caries prevalence is high [9].

The purpose of the present cross-sectional study was to estimate the caries status, with the incorporation of incipient caries, of 5-, 12-, and 15-year-old children and adolescents with an immigrant background in Greece, and to assess the relationship of their caries experience with various sociodemographic parameters. Furthermore, this study aimed to compare the immigrants’ caries status with that of the respective native Greek population and evaluate the effect of ethnic background on caries experience levels.

## 2. Materials and Methods

### 2.1. Study Sample

A sample of 707 5-, 12- and 15-year-old children and adolescents with immigrant backgrounds were selected during a national pathfinder survey (2013–2014), as part of a sampling procedure involving the selection of 3702 5-, 12- and 15-year-old subjects from the host population. Immigrant origin was designated if the participating subject (child or adolescent) or at least one of the parents was not born in Greece (individuals with a foreign background) [10]. Inclusion factors were the age of the subject (5, 12 or 15 years of age) and attending a public kindergarten, junior high school or high school, respectively. Special needs education schools were excluded from the sampling procedure. Specifically, the survey took place in public schools located in 24 sampling sites covering three urban municipalities with diverse socioeconomic backgrounds, which were selected randomly within each of the two bigger Greek metropolitan regions (Athens, the capital, and Thessaloniki, the co-capital), as well as one urban and one rural district selected randomly within each of six mainland counties and three islands [11,12]. Within each district, three schools, belonging either to the kindergarten, junior high school or high school academic level, were selected randomly, and within each school, samples of subjects attending kindergarten, the first year of junior high school or the first year of high school, respectively, were drawn by random classroom selection. At each sampling site, approximately 50 subjects from the host population were selected for each age group, as this is the standard size recommended as being appropriate by the pathfinder sampling methodology established by the WHO in populations with high disease levels [13]. Immigrant subjects were drawn from the same classrooms from which the host population’s sample was drawn; therefore, they were classmates of the Greek 5-, 12- and 15-year-olds. In total, 707 individuals with an immigrant background residing in urban and rural environments across the country were included in the survey, representing ~20% of the total population examined (4409 individuals), a proportion that was almost double than the proportion of immigrant public school students (~11%) in the total student population of Greece [2]. A minimum sample size of 382 subjects for each age group (191 Greeks and 191 immigrants) was calculated to have 90% statistical power to estimate a mean difference of at least 0.3 units in the number of decayed, missing and filled teeth between groups at the 95% confidence level, assuming unknown but equal variance across groups [14]. Based on data from previously published work [15], differences in caries experience levels between immigrant and Greek children were expected to be considerably higher.

### 2.2. Calibration

Before the clinical examinations, one of the co-authors (K.N.A) and nine other assigned examiners, who were qualified dental surgeons, were calibrated against an experienced academic clinical instructor who served as the ‘reference examiner’. At first, a theoretical session involving practical exercises with the aid of clinical depictions and extracted teeth with caries lesions was carried out; subsequently, a clinical examination of children and adolescents by the trainees and the reference examiner took place. In the clinical session of the calibration process, each of the 10 trainees performed two examinations of a group of 10, 12- and 15-year-old adolescents, who were also examined by the reference examiner. Ten 5-year-old children were initially examined by all trainees and the reference examiner, and subsequently re-examined by three of the trainees. The clinical examination procedure was aided by dental students who voluntarily served as chair-side assistants and were responsible for completing the examination sheets, by dental nurses, and by the information processing personnel, who carried out data analysis concurrently in the examination room. Hence, the monitoring of calibration performance was possible in real time, so that trainees’ testing and training could be repeated until at least acceptable scoring (i.e., fair agreement) was achieved by all participants. Calibration performance was measured at the tooth level, using the dmft/DMFT index and a cut-off diagnostic point at the d1 level (referring to Code 2 of the ICDAS II criteria). Inter-rater agreement, as shown by the weighted Cohen’s kappa, was estimated to be 0.55–0.91 and 0.63–0.76 for the 5-year-olds and the 12- and 15-year-olds, respectively, whereas the weighted percentage of agreement was >95% for all age groups. Moreover, the corresponding test/retest reliability statistic was calculated to be 0.63–0.90 and 0.31–0.89 for the 5-year-olds and the 12- and 15-year-olds, respectively, (for the 12- and 15-year-old age group, 9 out of the 10 examiners scored ≥0.53), whereas the weighted percentage of agreement was >92% for all age groups.

### 2.3. Clinical Examination

The subjects were examined inside the schools’ classrooms while sitting in reclining chairs, with the aid of appropriate portable lights, using dental mirrors and WHO periodontal probes in a standardised environment, as advocated by the World Health Organization. Moisture mitigation was achieved with the use of cotton rolls and gauze. Caries detection across all dental surfaces was performed visually, following the ICDAS-II classification criteria [16], after drying the teeth solely with gauze and not with a current of compressed air. Omitting the stage of air-drying is an acceptable methodological modification in epidemiological surveys; under these circumstances, caries lesions coded 1 and 2 according to the ICDAS system were added together and a composite (merged) code format was utilized [8].

### 2.4. Questionnaire Information

Information relevant to demographic and socioeconomic characteristics, as well as the dental health-related behavioural and dietary patterns of the examined population, was gathered through a questionnaire. Specifically, for the 5-year-old age group, the questionnaires were sent prior to the examination for completion at home by the parents/legal guardians of the children, whereas the 12- and 15-year-old adolescents filled out the questionnaires through a personal interview with the examiner at the time of the clinical examination. Whenever the interviewer had doubts about the validity of an adolescent’s answers relevant to the sociodemographic information that was provided, the parents were contacted to give additional information. The questionnaires were formatted in the Greek language and the information sought included the following areas of interest:

1. Sociodemographic characteristics: father’s and mother’s country of origin; years of stay in Greece of the father and the mother; country of student’s birth; gender of the student: male or female; location: urban or rural; father’s and mother’s level of education: (1) up to lower secondary, (2) upper secondary or non-university tertiary or (3) university.
2. Dental health-related behaviours: (A) 5-year-old age group: is toothbrushing performed on the child’s teeth? (no/yes). If yes, who brushes your child’s teeth? (1) The child without adult supervision; (2) the child supervised by an adult; (3) an adult. How often is toothbrushing performed? (1) Occasionally; (2) once per day; (3) twice per day. Is toothpaste used during toothbrushing? (no/yes). If yes, what kind of toothpaste is used? (1) Child; (2) adult. Up to what age did your child use a baby bottle? (1) 18 months; (2) 3 years; (3) >3 years. Your child was fed milk with a baby bottle (1) before he/she went for sleep, while he/she was still awake or (2) in his/her bed while sleeping. Did you clean the child’s teeth after baby bottle use? (no/yes). (B) For the 12- and the 15-year-old age groups: Do you brush your teeth and, if yes, how often? (1) Never; (2) occasionally/when I remember it; (3) once per day; (4) twice per day.
3. Dietary habits (for the 5-year-old age group): How often does your child consume the following: (1) sugary food, such as biscuits, cake and chocolate (rarely; 3–6 times per week; once per day; 2 or 3 times per day; >3 times per day); (2) sugary drinks such as packaged juice, chocolate milk and sugary milk (rarely; 3–6 times per week; once per day; 2 or 3 times per day; >3 times per day); (3) confectionary products, such as sugary chewing gums, lollipops and candies (rarely; 3–6 times per week; once per day; 2 or 3 times per day; >3 times per day).

### 2.5. Data Analysis

#### 2.5.1. Calculation of the Caries Indices

Primary clinical data recorded on individual examination sheets were processed and subsequently analysed. Caries experience was described by assigning ICDAS_0–6_ codes (i.e., code 0 = sound tooth surface; 1 = first visual change in enamel; 2 = distinct visual change in enamel; 3 = localized enamel breakdown, no dentine visible or underlying shadow; 4 = dentinal shadow, but not cavitation into dentine; 5 = distinct cavity with visible dentine; 6 = extensive distinct cavity with visible dentine involving at least half of the tooth surface) [16] to the d/D component of the dmf/DMF index formula. The following caries indices were estimated: the percentage of subjects who had not experienced caries on the d_3–6_mft/D_3–6_MFT level (d_3–6_mft/D_3–6_MFT = 0) as well as on the d_1–6_mft/D_1–6_MFT level (d_1–6_mft/D_1–6_MFT = 0), the percentage of subjects detected as having at least one tooth with incipient (enamel) caries: d_1–2_t/D_1–2_T ≥ 1, and the percentage of subjects needing restorative care: d_3–6_t/D_3–6_T ≥ 1. Moreover, the percentage of 5-year-old children with severe early childhood caries (S-ECC) was calculated (that is, dmfs ≥ 1 in the maxillary anterior teeth or dmfs ≥ 6) [17]. Moreover, calculation of initial caries scores (d_1–2_t/D_1–2_T), the d_3–6_mft/D_3–6_MFT index and its components (d_3–6_t/D_3–6_T, mt/MT and ft/FT) and the d_3–6_mfs/D_3–6_MFS index, was performed. In addition, the Care Index (CI) ft/d_3–6_mft, in %, or FT/D_3–6_MFT, in %, and the significant caries index (SiC) were calculated for each age group [18]. It should be noted that the “d/D” component of the dmft/dmfs and DMFT/DMFS indices refers to ICDAS codes 3–6, thus corresponding to caries lesions at the defect level [16], as the utilization of this merged index format in studies conducted in preschool children, schoolchildren and adolescents was found to minimize the difference in visual caries detection observed between the WHO and the ICDAS-II criteria. Therefore, the use of ICDAS code 3 as the cut-off diagnostic point of carious lesions needing restorative treatment presumably allows direct comparisons of d_3–6_mf/D_3–6_MF scores with previous studies that utilized the WHO criteria for visual caries detection (cut-off diagnostic point: obvious dentinal caries) in childhood populations [19,20].

#### 2.5.2. Independent Variables Description

Based on the data collected by the completed questionnaires, appropriate variables were created through further processing procedures. The following variables were selected and utilized for all age groups: gender (male/female), location (urban/rural), mother’s and father’s education (up to lower secondary, upper secondary or non-university tertiary, and university) and toothbrushing frequency (never or occasionally/once per day/twice per day). Additionally, solely for the 5-year-old age group, the following variables were also defined: subject who performs the toothbrushing (the child, unsupervised; the child, supervised by an adult; an adult), and frequency of consumption of sugary food, confectionary products or sugar-sweetened beverages (up to once per day; twice or more per day). Furthermore, the simplified debris index (DI-S) score (good: 0.0–0.6; fair: 0.7–1.8; poor: 1.9–3.0), which was calculated on the basis of the clinical recording of the proportion of buccal surface coverage of teeth 11, 31, 16 and 26, as well as of the lingual surface coverage of teeth 36 and 46 with soft debris [21], was utilized as an independent parameter.

#### 2.5.3. Statistical Analysis

A descriptive analysis of the caries prevalence and the caries experience indices within each immigrant age group was performed in association with the demographic characteristics (male or female gender, urban or rural location) and parental education level as a measure of the socioeconomic status of the subjects. Furthermore, negative binomial regression models were used to investigate the effect of parental education level on the d_3–6_mfs/D_3–6_MFS values of the immigrant children and adolescents. Specifically, the effect of the mother’s education level and the father’s education level was examined in separate analyses, each adjusted for gender, location, and father’s or mother’s education, respectively, as these parameters were considered to be possible confounders.

The mean values of the caries prevalence and the caries experience indices of the immigrant population were compared with the corresponding data of the host population. In order to explain any differences observed between the native Greek and immigrant groups more adequately, their sociodemographic characteristics (gender, location, parental education level) were compared. It should be noted that the caries indices and sociodemographic characteristics of the Greek 5-, 12- and 15-year-olds are presented and analysed elsewhere [11]. Moreover, to further explain any discrepancies in caries status observed between the immigrant and host populations, their dental health-related behavioural characteristics, such as their oral hygiene practices, their dietary patterns related to sugar consumption habits (available only for the 5-year-olds), and their oral hygiene levels were compared. Oral hygiene practices were described by the toothbrushing frequency and, for the 5-year-olds, by the subject who performs the toothbrushing. The dietary patterns of preschool children were described by the frequency of consumption of sugary food, confectionary products, and sugar-sweetened beverages, whereas the oral hygiene level of the examined population was described by the simplified debris index (DI-S) score and was categorized as good, fair or poor. It should be mentioned that the oral hygiene status (DI-S score) and oral hygiene practices of the Greek 12- and 15-year-old population have been presented and analysed elsewhere [12]. Finally, negative binomial regression models, crude or adjusted for potential confounding factors, specifically for the sociodemographic characteristics, toothbrushing frequency and the oral hygiene level of the subjects, were used for assessing the effect of ethnic background on the d_3–6_MFS/D_3–6_MFS scores of the total (immigrant and Greek) population.

Regarding missing data, no correction for non-participation or non-response was applied. For the descriptive analyses, the Mann–Whitney U and Kruskal–Wallis H non-parametric tests were applied to evaluate the differences between the mean values of the caries experience estimates, and Pearson’s chi-square test was utilized for assessing differences in proportion between caries prevalence estimates. To test the associations between the independent variables and the d_3–6_MFS/D_3–6_MFS outcomes, negative binomial regression analysis was selected, as the corresponding indices did not follow a Gaussian distribution: the data were positively skewed and demonstrated overdispersion. The estimated coefficient was the incidence rate ratio (IRR). In all regression models, comparisons within each independent variable were held against one of its categories that was set as a reference. Data processing and analysis was carried out in International Business Machines Corporation (IBM) SPSS Statistics, Armonk, New York, USA (PC version 26.0), and *p* ≤ 0.05 was the significance level accepted for all types of comparisons.

## 3. Results

### 3.1. Descriptive Analysis (Immigrant Population)

The sample of the examined subjects was 267, 234, and 206 in the 5-, 12-, and 15-year-old groups, respectively. The majority of children and adolescents with an immigrant background were born in Greece: specifically, 78.3%, 66.7% and 65.5% of the 5-, 12- and 15-year-olds, respectively. Furthermore, Albania was the dominant foreign country of parental origin for all age groups. Specifically, 43.8% of the fathers and 46.4% of the mothers of the 5-year-olds, 40.6% of the fathers and 41.5% of the mothers of the 12-year-olds, and 32.6% of the fathers and 32.1% of the mothers of the 15-year-olds were Albanian in origin, while other ethnicities, mostly from other Eastern European countries (such as Georgia, Romania, Bulgaria and Russia) followed at a considerable distance.

Table 1, Table 2 and Table 3 present the calculated percentages of subjects without a caries history, subjects demonstrating incipient caries, those who require restorative treatment and those who have received restorative care, as well as the mean initial caries levels and the d_3–6_mf/D_3–6_MF at the surface and tooth level (and its components) for the three age groups, according to urban/rural location, male/female gender, and parental education level.

The percentage of migrant children and adolescents with no caries history at the defect level (d_3–6_mft/D_3–6_MFT = 0) was found to be 41.6%, 33.3% and 32.5% for the 5-, 12-, and the 15-year-old age groups, respectively. By including initial lesions, the percentage of caries-free subjects (d_1–6_mft/D_1–6_MFT = 0) was reduced to 26.9%, 16.0%, and 12.6%, accordingly. Twelve-year-old immigrant adolescents whose parents had received more than a basic level of education were observed to have no caries experience at significantly higher proportions. Initial caries lesions were found in 15.4%, 17.5% and 19.9% of the 5-, 12-, and 15-year-old immigrant children and adolescents, respectively. Furthermore, mean initial caries estimates increased with age, being calculated at 1.1, 1.9 and 2.4 teeth, for the 5-, 12- and the 15-year-old immigrant subjects, accordingly.

In contrast, the mean caries experience at the defect level decreased from childhood through to adolescence, being estimated at 3.0 (d_3–6_mft), 2.4 (D_3–6_MFT) and 2.6 (D_3–6_MFT) for the 5-, 12- and 15-year-old migrant subjects, respectively. Additionally, 12-year-old adolescents with better-educated parents and 15-year-old adolescents with better-educated mothers presented with significantly lower mean D_3–6_MFT/S and D_3–6_T scores. Around half of the subjects of all ages demonstrated at least one carious tooth needing restorative treatment (56.6% of the 5-year-olds, 53.4% of the 12-year-olds and 49.5% of the 15-year-olds), whereas approximately one-third of the 5-year-olds (31.8%) presented with severe early childhood caries (S-ECC). Migrant adolescents with less educated parents demonstrated higher restorative treatment needs. Moreover, a lower parental education level was more common among 5-year-old migrants presenting with S-ECC. The Care Index was very low for the 5-year-olds (8.2%), whereas the corresponding 12- and 15-year-olds’ indices were moderately higher (30.2% and 39.1%, respectively). Among the 5-year-olds, girls had satisfied their dental restorative needs at a higher proportion. Moreover, the significant caries indices (SiC) of the migrant population were 7.4, 5.2 and 6.1 for the 5-, 12- and 15-year-olds, respectively.

### 3.2. Negative Binomial Regression Analysis (Immigrant Population)

Negative binomial regression analysis of the effect of parental education on the caries experience levels (d_3–6_mfs/D_3–6_MFS) of the immigrant population (Table 4) demonstrated maternal education level to be a significant parameter for the adolescent age groups. Specifically, 12- and 15-year-old immigrants whose mothers demonstrated a lower educational level exhibited a considerably elevated probability of having higher caries experience levels than their peers with university-educated mothers (IRR = 1.85; *p* = 0.025, and IRR = 2.40; *p* < 0.001, for mothers of 12- and 15-year-olds, respectively, with basic education, and IRR = 1.88; *p* = 0.006, for mothers of 15-year-olds with middle-level education).

### 3.3. Comparison with the Host Population

#### 3.3.1. Caries Indices

**Figure 1:**
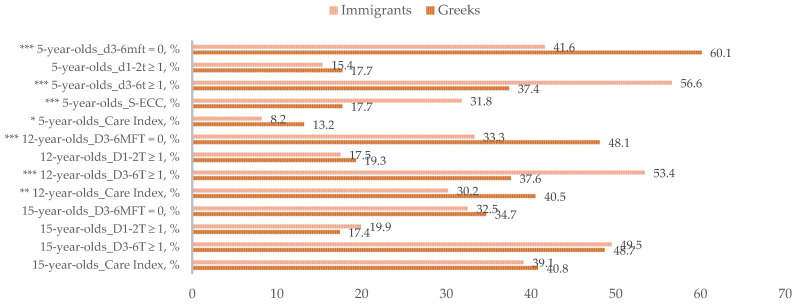
Caries prevalence of 5-, 12- and 15-year-old children and adolescents of immigrant origin in Greece, compared with their Greek age-mates. Percentage (%) with no caries experience at the defect level (d_3–6_mft/D_3–6_MFT = 0), percentage (%) with initial caries (d_1–2_t/D_1–2_T ≥ 1), percentage (%) in need of treatment (d_3–6_t/D_3–6_T ≥ 1), percentage (%) of 5-year-olds with severe early childhood caries (S-ECC) and Care Index (%) at the tooth level. * *p* ≤ 0.05, ** *p* ≤ 0.01 and *** *p* ≤ 0.001, as analysed by Pearson’s chi-square test and the Mann–Whitney U-test (for the Care Index analysis). Caries estimates of the Greek 5-, 12- and 15-year-olds were adapted from Diamanti et al. (2021) [11].

**Figure 2:**
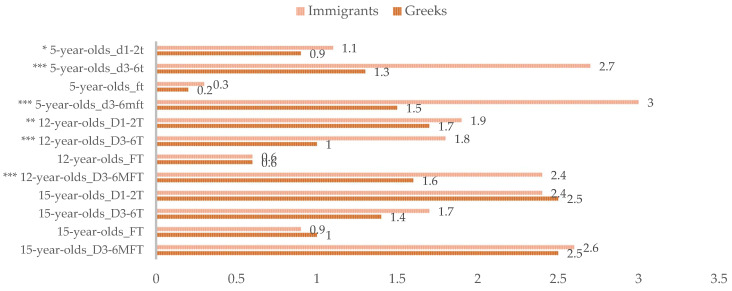
Caries experience of 5-, 12- and 15-year-old children and adolescents of immigrant origin in Greece, compared with their Greek age-mates. Mean ICDAS_1–2_ at the tooth level (d_1–2_t/D_1–2_T), mean d_3–6_mft/D_3–6_MFT, mean d_3–6_t/D_3–6_T and mean ft/FT. * *p* ≤ 0.05, ** *p* ≤ 0.01 and *** *p* ≤ 0.001, as analysed by the Mann–Whitney U-test. Caries estimates of the Greek 5-, 12- and 15-year-olds were adapted from Diamanti et al. (2021) [11].

Figure 1 and Figure 2 give a schematic comparison of the caries prevalence and the caries experience indices between the immigrant and the host populations. It should be noted that the host population included 3702 subjects in total, specifically, 1222, 1252 and 1228 5-, 12- and 15-year-old children and adolescents, respectively. For the 5- and 12-year-old groups, immigrant subjects had not experienced caries at considerably lower proportions than their Greek peers. Furthermore, higher proportion of 5- and 12-year-old individuals in the immigrant groups presented with at least one carious tooth needing restorative treatment; similarly, a substantially higher proportion of migrant 5-year-olds was found to suffer from severe early childhood caries compared with the host population. Moreover, mean D_3–6_MFT and D_3–6_T indices were considerably higher in the migrant than in the host 5- and 12-year-olds. Moreover, the coverage of dental restorative needs was lower in the immigrant than in the host populations of 5- and 12-year-olds. Regarding the prevalence of early carious lesions, no considerable differences were observed between 5- and 12-year-old immigrant and host subjects. However, mean initial caries estimates were significantly higher in the migrant population. Considering the 15-year-old adolescents, no significant differences between immigrant and host populations were observed in any of the caries prevalence, caries experience or restorative care indices.

#### 3.3.2. Sociodemographic and Behavioural Parameters

In Table 5, the distribution of the immigrant age groups according to gender, location, parental education level, oral hygiene practices, oral hygiene level and dietary practices (available only for the 5-year-olds), in comparison with their host peers, is presented. The proportion of male and female subjects was similar in both the immigrant and the native Greek age-groups. Higher percentages of 5- and 15-year-old immigrants resided in urban than in rural areas, compared with their host peers (*p* < 0.001). In all age groups, parental education attainment was considerably lower in the immigrant than in the host population. Among the 5-year-old children, a higher proportion of immigrants performed toothbrushing by themselves unsupervised by an adult and also demonstrated worse oral hygiene levels than their host peers (*p* < 0.001). Moreover, a higher proportion of immigrant preschool children frequently consumed (at least twice per day) confectionery products and sugar-sweetened beverages than their Greek peers (*p* < 0.001). Similarly, among the 12-year-olds, a higher percentage of immigrants performed toothbrushing less than twice per day (*p* = 0.033) and also demonstrated poorer oral hygiene maintenance (*p* = 0.002) compared with their host peers. No considerable differences were found among the 15-year-old adolescents of either immigrant or native Greek origin, in terms of toothbrushing frequency (*p* = 0.643) and oral hygiene status (*p* = 0.173).

#### 3.3.3. Negative Binomial Regression Analysis (Total, Immigrant and Greek Population)

Negative binomial regression modelling of the effect of the ethnic background on the d_3–6_mfs and the D_3–6_MFS indices (Table 6), either in crude models or after controlling for the sociodemographic characteristics, toothbrushing frequency and oral hygiene level of the total (immigrant and Greek) population, showed that having a ‘foreign background’ considerably increased the likelihood of demonstrating higher caries experience levels for the 5- and the 12-year-old subjects (IRR = 1.61; *p* = 0.001, and IRR = 1.27; *p* = 0.006, respectively, compared with having a native Greek background in the adjusted models), whereas no considerable ethnic difference was found for the 15-year-old subjects (IRR = 1.09; *p* = 0.357 for the crude model and IRR = 1.10; *p* = 0.331 for the adjusted model).

## 4. Discussion

The purpose of the present epidemiological survey was to determine the caries status of 5-, 12-, and 15-year-old children and adolescents of the ‘settled’ immigrant population in Greece, with the inclusion of initial caries lesions, and to evaluate the levels of disease in association with sociodemographic characteristics. The study also aimed to compare the immigrants’ caries levels with those of their host peers and assess the effect of ethnic background on caries experience levels. The ultimate goal was to support health authorities to organize oral health services and develop more transculturally targeted educational and preventive programmes to positively impact the oral health of immigrant populations.

The present study is the first to attempt to depict a representative picture of the dental health status of the urban and rural immigrant child population in Greece; other investigations included only residents of one prefecture (within Attica, the capital region), and reported that possessing an immigrant background considerably increased the probability of poorer oral health outcomes in children up to 12 years of age [15,22].

As has been mentioned, the majority of ‘settled’ immigrants in Greece originate from Eastern European countries, most frequently from Albania. Epidemiological evidence from studies conducted in Western European countries suggests that, among adults, adolescents and children with an immigrant background, those with Eastern European origin present a higher dental caries prevalence [5,23,24,25,26,27]. Moreover, native Eastern European children and adolescents present with considerably higher caries levels compared with their Western European peers [11], an observation that is possibly associated with the privatisation and decentralisation of the oral healthcare delivery system in these territories during the last three decades, which has impeded the provision of dental care services in childhood populations, leading to detrimental effects on disease levels [28]. For example, 5- and 12-year-old inhabitants of Albania, which is the dominant source country of immigrants in Greece, demonstrated considerably high mean dmft/DMFT values, (4.4 and 3.7, respectively), very low percentages of caries-free children at the cavitation level (16% and 13%, respectively) and very high levels of untreated caries (80% and 70%, respectively) [29,30]. Compared with other Eastern European inhabitant childhood populations, Greek children appear to be in a markedly optimal position in terms of caries prevalence and caries experience scores [11]; in addition, between 2004 and 2014, a small-to-moderate improvement in their dental health status was observed [11,31].

In the present study, poorer dental caries status of the 5- and 12-year-old children and adolescents with an immigrant background was observed, compared with their Greek peers. Fewer caries-free subjects, higher proportions of subjects with untreated caries—particularly preschool children experiencing severe early childhood caries—worse caries experience figures, including initial caries lesions, and less coverage of the dental restorative needs were recorded in the immigrant population than in their native Greek age-mates. However, at age 15, the differences in caries prevalence and experience estimates, and in the levels of restorative care were considerably reduced, such that no considerable variation was observed between the subjects with immigrant and host backgrounds.

It can be argued that dental health until early adolescence is presumably driven by parental knowledge, attitudes and beliefs regarding dietary habits, oral hygiene practices, dental visits and treatment motifs and patterns, since parents have a leading role and decisive impact on the environment in which children of younger age groups are raised [32]. The significance that the primary caregivers attribute to dental health is often formed by their socioeconomic status and cultural norms, which subsequently influence attitudes, beliefs and oral health-related practices and patterns of behaviour [33]. In the present study, the parental education level, a component factor characterizing the socioeconomic status of the family, was observed to be considerably lower in the immigrant than in the host population. The education level of parents is considered to be indicative of the material wealth and intellectual background of the family [10]. It has been shown to be positively associated with their level of knowledge regarding their children’s dental health maintenance [32], may determine their interest in and willingness to benefit from oral health promotion-oriented actions such as preventive interventions and oral health education and awareness-raising programmes [32], and has been identified as being a strong caries risk indicator throughout childhood and adolescence [34,35]; however, it generally does not suffice to fully explain the inequality in oral health outcomes observed between immigrant and host West European childhood populations [10,36]. Additional mechanisms involving individual, household, societal or policy dimensions, such as the reasons for relocation in the foreign country, the frequency of encountering prejudice and discrimination, and the different cultural standards, may also play a dominant role in the development of oral health disparities [36].

Therefore, among caries determinants, not only socioeconomic status but also sociocultural conditions and the associated oral health-related behaviours and habits may play significant roles [37]. Epidemiological studies capturing immigrant populations in the European area revealed that dental hygiene routines (e.g., toothbrushing), the amount of fermentable carbohydrate consumption, and the level of oral health awareness and personal perspectives towards oral health maintenance were, in general, unfavourable among migrants in comparison with native populations [5]. Moreover, the low importance of oral health maintenance of their children was observed to be an attribute of the majority of immigrant parents; furthermore, insufficient language skills, less adherence to supervised oral hygiene practice, and the adoption of cariogenic dietary patterns without taking their negative impact on dental health into account were reasons reported to be significant barriers, producing unfavourable dental health results [5]. Moreover, costly dental services, a lack of information about the available options for the fulfilment of dental treatment needs, and different attitudes and belief systems from the host population seem to have created considerable obstacles to oral health services utilization [5]. The results of the present study corroborate these observations, as, among the 5- and 12-year-old subjects, suboptimal toothbrushing practices and insufficient oral hygiene maintenance were more common in the immigrant than in the Greek population. Furthermore, higher proportions of immigrant preschool children followed caries-promoting dietary patterns than their Greek peers.

Therefore, the increased caries levels that were observed among the 5- and 12-year-old immigrant children and adolescents compared with the host population can be attributed to their lower socioeconomic position, as described by parental education level; to the adoption of adverse oral hygiene behaviours, as outlined by their toothbrushing patterns; to their worse oral hygiene maintenance, as defined by the DI-S score; and, for the 5-year-olds for whom data were available, to caries-promoting dietary habits. It should be noted that in Greece, fulfilment of dental treatment needs and preventive procedures is performed primarily by the costly private sector. Public dental health coverage includes all children and adolescents up to 18 years of age, irrespective of whether they are of Greek nationality or not, or whether they are legal or illegal residents; however, the net of public dental care centres under the national health system has not been adequately expanded, and although school-based oral examinations, particularly focusing on primary education-level students, are being organized sporadically, public dental services mainly include basic emergency-oriented treatment.

Considering the 15-year-olds with an immigrant background, no substantial difference in dental caries status was observed compared with the Greek 15-year-olds. In European countries, research into the dental health status of adolescent immigrants in relation to their host peers has been inconclusive [5]. Epidemiological studies from Sweden [25,38], Germany [26] and Spain [39] have shown considerably increased caries levels among adolescents with an immigrant origin, in comparison with their native peers. Higher caries experience was also found among adolescents of foreign origin in Denmark in comparison with their Danish peers, but the difference was smaller than that observed between younger immigrant and host age groups [37]. Furthermore, in Britain, a weaker association between ethnic background and dental caries levels was documented among 15-year-old adolescents than in 5-year-old children [40]. Adolescence is a landmark transition period, where family impact gradually diminishes, whereas openness to influences from the broader social environment, such as school, peer groups, mass media and youth culture, increases considerably [41]. As a result, oral health maintenance-associated behaviours may alter during adolescence, such as toothbrushing practices and dietary patterns, and this reset of attitudes and priorities may lead to a more balanced picture in terms of dental health between adolescents with immigrant and host backgrounds [40]. In the present study, no considerable difference was found between the 15-year-old adolescents of immigrant and native Greek origin, in terms of the frequency of toothbrushing and the level of oral hygiene observed.

The results of the present study identify an immigrant background as considerably increasing the risk of higher caries experience levels in childhood and early adolescence, similar to other European studies [5]. After controlling for the effect of sociodemographic characteristics, oral hygiene practices and oral hygiene level, 5- and 12-year-olds with a foreign background still had an elevated probability of higher caries experience levels than their host peers. This implies that the category ‘immigrant background’ encompasses additional cultural dimensions associated with caries, which act independently of the sociobehavioural and clinical explanatory variables included in the present analysis. For example, in the present pathfinder survey, 5-year-old children with an immigrant background were found to have a history of nocturnal baby bottle feeding at a higher proportion than their Greek peers (Appendix A Table A1).

In the present study, maternal education level served as a significant predictor of caries experience levels among immigrant 12- and 15-year-olds, but not among the 5-year-old age immigrant group. Specifically, 12- and 15-year-old immigrant adolescents with less educated mothers exhibited an increased probability of demonstrating higher caries experience levels compared with their peers with university educated mothers. Maternal education level was reported as being the most important indicator of family socioeconomic status in association with dental caries in Dutch schoolchildren [42]. Furthermore, previous studies have shown that maternal education level exhibits stronger associations with caries experience in young children than in adolescent populations [43]; nevertheless, the present study reported the opposite. This finding can be potentially attributed to factors related to the young age of the children, such as the possible lack of cooperation, which may have impeded the effectiveness of oral hygiene practices and may have had an adverse impact on the frequency and efficacy of dental visits by the 5-year-old immigrant children.

The present study was the first in Greece with the aim of reporting the caries status of children and adolescents with an immigrant background residing in urban and rural areas across the country, and comparing their caries scores with their Greek peers. Another strength of the study was that the clinical recordings included not only caries lesions at the defect level but also incipient caries, according to the ICDAS II criteria. However, one limitation was that the clinical examinations were performed in the school classrooms without the use of compressed air for tooth cleaning and drying; therefore, some ICDAS code 1 lesions, specifically those that might have not picked up stains from the oral environment, may have escaped detection. Another limitation was that the sample of the immigrant subjects (*n* = 707) was considerably smaller than the sample of their Greek counterparts (*n* = 3702); however, it was almost double the proportion of immigrant public school students (children and adolescents) (~11%) in the total student population of Greece, and was estimated to be adequate for detecting differences in caries experience levels. Moreover, the proportion of urban and rural subjects differed significantly between the immigrant and the host 5- and 15-year-olds, potentially producing some degree of bias into the group comparisons; nevertheless, caries scores were not found to differ significantly between rural and urban immigrant 5- and 15-year-old participants. Furthermore, the general health status of the subjects included in the present study was not recorded, which theoretically may have resulted in systemic bias; however, subjects were drawn from the general and presumably healthy student population; for example, children and adolescents attending special needs schools were excluded from the sampling procedure.

## 5. Conclusions

In conclusion, 5- and 12-year-old subjects with an immigrant background demonstrated considerably increased dental caries levels and more unfulfilled treatment demands than their native Greek age-mates. No substantial ethnic differences in dental health were found among 15-year-old adolescents. The strength of the association between ethnic background and caries experience levels attenuated gradually with increasing age. Among the immigrants, no association was observed among caries experience in the primary dentition and parental education level, whereas for caries in the permanent dentition, only maternal education level could be identified as a prominent indicator. Oral health promotion strategies and preventive programmes should be organised and implemented, particularly during the preschool and primary school years, focusing on underprivileged childhood populations with an immigrant origin, with the aim of reducing ethnic disparities in oral health.

## Figures and Tables

**Table 1:** Caries prevalence and caries experience of 5-year-old children of immigrant origin in Greece. Number of children (*n*), percentage of caries-free children on the d_3–6_mft level (d_3–6_mft = 0), percentage of caries-free children on the d_1–6_mft level (d_1–6_mft = 0), percentage of children with initial caries (d_1–2_t ≥ 1), percentage of children in need of restorative treatment (d_3–6_t ≥ 1), percentage of children with severe early childhood caries (S-ECC), mean ICDAS_1–2_ on the tooth level (d_1–2_t) mean d_3–6_mft and its components (d_3–6_t, mt, ft), mean d_3–6_mfs and Care Index (CI, %) on the tooth level, according to the area of residence, gender and parents’ educational level.

	*n* ^†^	d_3–6_mft = 0 *n* (%)	d_1–6_mft = 0 *n* (%)	d_1–2_t ≥ 1 *n* (%)	d_3–6_t ≥ 1 *n* (%)	S-ECC *n* (%)	d_1–2_t (±SD)	d_3–6_mft (±SD)	d_3–6_t (±SD)	mt (±SD)	ft (±SD)	d_3–6_mfs (±SD)	CI *n* (%)
Area of residence													
Rural	70	33 (47.1)	20 (28.6)	13 (18.6)	34 (48.6)	24 (34.3)	1.2 (1.6)	2.8 (4.0)	2.4 (4.0)	0.1 (0.8)	0.4 (1.2)	5.2 (8.8)	10 (14.7)
Urban	197	78 (39.6)	50 (25.4)	28 (14.2)	117 (59.4)	61 (31.0)	1.0 (1.5)	3.1 (3.7)	2.8 (3.6)	0.0 (0.1)	0.2 (1.1)	4.9 (7.6)	12 (6.2)
Gender													
Male	137	56 (40.9)	38 (27.7)	18 (13.1)	80 (58.4)	43 (31.4)	1.1 (1.6)	3.2 (4.1)	3.0 (3.9)	0.0 (0.0)	0.2 (1.1)	5.5 (8.9)	6 (4.6) *
Female	130	55 (42.3)	32 (24.6)	23 (17.7)	71 (54.6)	42 (32.3)	1.1 (1.5)	2.8 (3.5)	2.4 (3.4)	0.1 (0.6)	0.4 (1.2)	4.5 (6.6)	16 (12.2)
Father’s education													
≤Lower secondary	98	35 (35.7)	21 (21.4)	14 (14.3)	61 (62.2)	39 (39.8) *	1.1 (1.4)	3.5 (4.3)	3.4 (4.2)	0.0 (0.1)	0.1 (0.5)	6.1 (8.9)	6 (5.9)
Upper secondary or non-university tertiary	120	52 (43.3)	34 (28.3)	18 (15.0)	65 (54.2)	31 (25.8)	1.1 (1.7)	2.6 (3.3)	2.2 (3.2)	0.1 (0.6)	0.4 (1.3)	4.0 (6.5)	13 (11.0)
University	31	18 (58.1)	13 (42.0)	5 (16.1)	13 (41.9)	7 (22.6)	1.0 (1.4)	2.1 (3.4)	1.7 (2.3)	0.0 (0.0)	0.4 (2.0)	4.1 (9.1)	2 (6.9)
Mother’s education													
≤Lower secondary	89	33 (37.1)	23 (25.8)	10 (11.2)	54 (60.7)	37 (41.6) *	1.0 (1.4)	3.7 (4.4)	3.6 (4.4)	0.1 (0.7)	0.1 (0.6)	6.0 (8.6)	6 (6.6)
Upper secondary or non-university tertiary	119	53 (44.5)	36 (30.3)	17 (14.3)	64 (53.8)	31 (26.1)	1.1 (1.7)	2.4 (3.2)	2.2 (3.1)	0.0 (0.0)	0.2 (1.0)	4.1 (6.6)	10 (8.5)
University	43	21 (48.8)	10 (23.3)	11 (25.6)	21 (48.8)	8 (18.6)	1.3 (1.7)	2.2 (3.4)	1.7 (2.6)	0.0 (0.0)	0.6 (2.1)	4.3 (9.2)	5 (12.5)
Total	267	111 (41.6)	70 (26.2)	41 (15.4)	151 (56.6)	85 (31.8)	1.1 (1.6)	3.0 (3.8)	2.7 (3.9)	0.0 (0.4)	0.3 (1.1)	5.0 (7.9)	22 (8.2)

^†^ Number of observations may be less than the total for some variables due to missing data. The symbol (*) within each column subset indicates that the included values differ at the *p* ≤ 0.05 level, as analysed by the Mann–Whitney U-test, the Kruskal–Wallis H-test and Pearson’s chi-square test.

**Table 2:** Caries prevalence and caries experience of 12-year-old children of immigrant origin in Greece. Number of children (*n*), percentage of caries-free children on the D_3–6_MFT level (D_3–6_MFT = 0), percentage of caries-free children on the D_1–6_MFT level (D_1–6_MFT = 0), percentage of children with initial caries (D_1–2_T ≥ 1), percentage of children in need of restorative treatment (D_3–6_T ≥ 1), mean ICDAS_1–2_ on the tooth level (D_1–2_T), mean D_3–6_MFT and its components (D_3–6_T, MT, FT), mean D_3–6_MFS and Care Index (CI, %) on the tooth level, according to the area of residence, gender and parents’ educational level.

	*n* ^†^	D_3–6_MFT = 0 *n* (%)	D_1–6_MFT = 0 *n* (%)	D_1–2_T ≥ 1 *n* (%)	D_3–6_T ≥ 1 *n* (%)	D_1–2_T (±SD)	D_3–6_MFT (±SD)	D_3–6_T (±SD)	MT (±SD)	FT (±SD)	D_3–6_MFS (±SD)	CI *n* (%)
Area of residence												
Rural	66	21 (31.8)	8 (12.5)	13 (19.7)	34 (51.5)	2.1 (2.7)	2.7 (2.8)	1.8 (2.6)	0.1 (0.2)	0.6 (1.2)	4.5 (5.7)	24 (35.7)
Urban	168	57 (33.9)	29 (17.4)	28 (16.7)	91 (54.2)	1.8 (1.7)	2.4 (2.4)	1.7 (2.3)	0.0 (0.1)	0.6 (1.2)	3.3 (3.7)	47 (28.0)
Gender												
Male	114	42 (36.8)	20 (17.7)	22 (19.3)	56 (49.1)	1.6 (1.8)	2.2 (2.4)	1.6 (2.3)	0.0 (0.0)	0.5 (1.1)	3.2 (4.0)	33 (29.3)
Female	118	35 (29.7)	16 (13.8)	19 (16.1)	68 (57.6)	2.1 (2.3)	2.7 (2.6)	1.9 (2.4)	0.0 (0.2)	0.7 (1.3)	4.0 (4.7)	37 (31.2)
Father’s education												
≤Lower secondary	107	25 (23.4) *	7 (6.7) *	18 (16.8)	68 (63.6) *	1.9 (1.8)	3.1 (2.8) *	2.4 (2.7) *	0.0 (0.1)	0.7 (1.3)	4.8 (5.3) *	28 (26.6)
Upper secondary or non-university tertiary	86	35 (40.7)	18 (20.9)	17 (19.8)	40 (46.5)	2.0 (2.4)	2.0 (2.2)	1.3 (1.8)	0.0 (0.2)	0.6 (1.2)	2.7 (3.3)	28 (32.3)
University	36	16 (44.4)	11 (30.6)	5 (13.9)	15 (41.7)	1.5 (1.9)	1.6 (1.8)	1.0 (1.6)	0.0 (0.0)	0.5 (1.2)	2.1 (2.2)	14 (37.9)
Mother’s education												
≤Lower secondary	108	24 (22.2) *	9 (8.6) *	15 (13.9)	69 (63.9) *	1.8 (1.9)	3.1 (2.7) *	2.3 (2.7) *	0.0 (0.2)	0.6 (1.2)	4.9 (5.2) *	32 (29.2)
Upper secondary or non-university tertiary	83	33 (39.8)	13 (15.7)	20 (24.1)	41 (49.4)	2.3 (2.4)	2.0 (2.2)	1.4 (1.8)	0.0 (0.1)	0.7 (1.2)	2.7 (3.3)	20 (24.5)
University	39	20 (51.3)	14 (35.9)	6 (15.4)	13 (33.3)	1.4 (1.7)	1.5 (2.0)	0.9 (1.7)	0.0 (0.0)	0.5 (1.2)	2.0 (2.5)	19 (47.8)
Total	234	78 (33.3)	37 (16.0)	41 (17.5)	125 (53.4)	1.9 (2.1)	2.4 (2.5)	1.8 (2.4)	0.0 (0.1)	0.6 (1.2)	3.6 (4.4)	71 (30.2)

^†^ Number of observations may be less than the total for some variables due to missing data. The symbol (*) within each column subset indicates that the included values differ at the *p* ≤ 0.05 level, as analysed by the Mann–Whitney U-test, the Kruskal–Wallis H-test and Pearson’s chi square test.

**Table 3:** Caries prevalence and caries experience of 15-year-old children of immigrant origin in Greece. Number of children (*n*), percentage of caries-free children on the D_3–6_MFT level (D_3–6_MFT = 0), percentage of caries-free children on the D_1–6_MFT level (D_1–6_MFT = 0), percentage of children with initial caries (D_1–2_T ≥ 1), percentage of children in need of restorative treatment (D_3–6_T ≥ 1), mean ICDAS_1–2_ on the tooth level (D_1–2_T), mean D_3–6_MFT and its components (D_3–6_T, MT, FT), mean D_3–6_MFS and Care Index (CI, %) on the tooth level, according to the area of residence, gender and parents’ educational level.

	*n* ^†^	D_3–6_MFT = 0 *n* (%)	D_1–6_MFT = 0 *n* (%)	D_1–2_T ≥ 1 *n* (%)	D_3–6_T ≥ 1 *n* (%)	D_1–2_T (±SD)	D_3–6_MFT (±SD)	D_3–6_T (±SD)	MT (±SD)	FT (±SD)	D_3–6_MFS (±SD)	CI *n* (%)
Area of residence												
Rural	40	13 (32.5)	3 (7.5)	10 (25.0)	18 (45.0)	2.8 (4.0)	2.8 (2.9)	1.7 (2.4)	0.1 (0.3)	1.1 (1.7)	3.7 (4.1)	19 (47.5)
Urban	166	54 (32.5)	23 (13.9)	31 (18.7)	84 (50.6)	2.3 (2.7)	2.5 (2.8)	1.6 (2.4)	0.0 (0.2)	0.9 (1.5)	3.7 (5.0)	61 (37.0)
Gender												
Male	87	30 (34.5)	15 (17.2)	15 (17.2)	44 (50.6)	2.0 (2.6)	2.3 (2.8)	1.7 (2.5)	0.1 (0.2)	0.6 (1.1)	3.5 (5.0)	29 (32.8)
Female	119	37 (31.1)	11 (9.2)	26 (21.8)	58 (48.7)	2.7 (3.2)	2.7 (2.9)	1.6 (2.4)	0.0 (0.2)	1.1 (1.8)	3.9 (4.6)	52 (43.4)
Father’s education												
≤Lower secondary	52	17 (32.7)	5 (9.6)	12 (23.1)	30 (57.7) *	2.7 (3.2)	2.5 (2.9)	1.8 (2.6)	0.1 (0.3)	0.7 (1.4)	3.6 (4.4)	14 (27.8)
Upper secondary or non-university tertiary	101	30 (29.7)	15 (14.9)	15 (14.9)	53 (52.5)	2.2 (2.8)	2.8 (2.9)	1.8 (2.5)	0.0 (0.2)	1.0 (1.5)	4.1 (5.3)	39 (39.1)
University	49	19 (38.8)	6 (12.2)	13 (26.5)	17 (34.7)	2.6 (3.2)	2.2 (2.8)	1.3 (2.2)	0.0 (0.0)	1.0 (1.7)	3.1 (4.3)	26 (52.8)
Mother’s education												
≤Lower secondary	55	13 (23.6)	4 (7.3)	9 (16.4)	33 (60.0) *	2.2 (2.7)	3.2 (3.0) *	2.1 (2.8) *	0.1 (0.3)	1.1 (1.6)	4.7 (4.6) *	20 (37.2)
Upper secondary or non-university tertiary	80	26 (32.5)	11 (13.8)	15 (18.8)	44 (55.0)	2.5 (3.0)	2.7 (3.0)	1.9 (2.5)	0.0 (0.2)	0.9 (1.6)	4.3 (5.7)	25 (31.1)
University	70	28 (40.0)	11 (15.7)	17 (24.3)	24 (34.3)	2.4 (3.2)	1.9 (2.4)	1.0 (1.9)	0.0 (0.1)	0.8 (1.4)	2.3 (3.4)	36 (52.1)
Total	206	67 (32.5)	26 (12.6)	41 (19.9)	102 (49.5)	2.4 (3.0)	2.6 (2.9)	1.7 (2.4)	0.0 (0.2)	0.9 (1.5)	3.7 (4.8)	81 (39.1)

^†^ Number of observations may be less than the total for some variables due to missing data. The symbol (*) within each column subset indicates that the included values differ at the *p* ≤ 0.05 level, as analysed by the Mann–Whitney U-test, the Kruskal–Wallis H-test and Pearson’s chi square test.

**Table 4:** Effect of parental education level on the d_3–6_mfs/D_3–6_MFS of the 5-, 12- and 15-year-old children of immigrant origin in Greece (negative binomial regression analyses).

		Independent Variable				Independent Variable			
		Mother’s Education Level (Ref. University)				Father’s Education Level (Ref. University)			
Dependent variable	d_3–6_mfs	5-year-olds (*n* = 248)				5-year-olds (*n* = 248)			
		B	IRR ^†^	95% C.I.	*p*-Value	B	IRR ^‡^	95% C.I.	*p*-Value
		−0.001	1.00 ^1^	(0.48. 2.07)	0.998	−0.002	1.00 ^1^	(0.42. 2.40)	0.997
		0.216	1.24 ^2^	(0.55. 2.80)	0.604	0.291	1.34 ^2^	(0.51. 3.52)	0.556
Dependent variable	D_3–6_MFS	12-year-olds (*n* = 227)				12-year-olds (*n* = 227)			
		B	IRR ^†^	95% C.I.	*p*-Value	B	IRR ^‡^	95% C.I.	*p*-Value
		0.262	1.30 ^1^	(0.76. 2.22)	0.337	0.090	1.09 ^1^	(0.67. 1.79)	0.719
		0.614	1.85 ^2^	(1.08. 3.17)	0.025	0.444	1.56 ^2^	(0.96. 2.55)	0.076
Dependent variable	D_3–6_MFS	15-year-olds (*n* = 202)				15-year-olds (*n* = 202)			
		B	IRR ^†^	95% C.I.	*p*-Value	B	IRR ^‡^	95% C.I.	*p*-Value
		0.631	1.88 ^1^	(1.20. 2.95)	0.006	0.034	1.04 ^1^	(0.64. 1.69)	0.890
		0.877	2.40 ^2^	(1.56. 3.71)	<0.001	−0.281	0.76 ^2^	(0.46. 1.25)	0.276

^†^ Model adjusted for gender, location and father’s education. ^‡^ Model adjusted for gender, location and mother’s education. ^1^ Upper secondary or non-university tertiary, ^2^ ≤lower secondary. IRR: incidence rate ratio.

**Table 5:** Distribution of 5-, 12- and 15-year-old children and adolescents of immigrant and of native Greek origin according to location, gender, parental education level, oral hygiene practices, oral hygiene level (DI-S score) and dietary patterns (data available only for preschool children).

	5-Year-Olds			12-Year-Olds			15-Year-Olds		
	Greeks	Immigrants	*p*-Value ^‡^	Greeks	Immigrants	*p*-Value ^‡^	Greeks	Immigrants	*p*-Value ^‡^
	(*n* = 1222)	(*n* = 267)		(*n* = 1252)	(*n* = 234)		(*n* = 1228)	(*n* = 206)	
Area of residence *n* ^†^ (%)									
Rural	463 (37.9)	70 (26.2)	<0.001	426 (34.0)	66 (28.2)	0.096	438 (35.7)	40 (19.4)	<0.001
Urban	759 (62.1)	197 (73.8)		826 (66.0)	168 (71.8)		790 (64.3)	166 (80.6)	
Gender *n* ^†^ (%)									
Male	595 (48.7)	137 (51.3)	0.458	612 (49.2)	114 (49.1)	1.000	569 (46.4)	87 (42.2)	0.29
Female	627 (51.3)	130 (48.7)		631 (50.8)	118 (50.9)		658 (53.6)	119 (57.8)	
Father’s education *n* ^†^ (%)									
≤Lower secondary	193 (16.0)	98 (39.4)		286 (22.9)	107 (46.7)		276 (22.5)	52 (25.7)	
Upper secondary/	738 (61.3)	120 (48.2)	<0.001	580 (46.5)	86 (37.6)	<0.001	545 (44.5)	101 (50.0)	0.049
non-university tertiary									
University	272 (22.6)	31 (12.4)		381 (30.6)	36 (15.7)		403 (32.9)	49 (24.3)	
Mother’s education *n* ^†^ (%)									
≤Lower secondary	94 (7.8)	89 (35.5)		236 (18.9)	108 (47.0)		213 (17.4)	55 (26.8)	
Upper secondary/	774 (64.0)	119 (47.4)	<0.001	612 (49.1)	83 (36.1)	<0.001	590 (48.1)	80 (39.0)	0.003
non-university tertiary									
University	342 (28.3)	43 (17.1)		399 (32.0)	39 (17.0)		423 (34.5)	70 (34.1)	
DI-S score *n* ^†^ (%)									
Good (0.0–0.6)	671 (54.9)	93 (34.8)	<0.001	549 (43.9)	79 (33.8)	0.002	698 (56.9)	103 (50.0)	0.173
Fair (0.7–1.8)	525 (43.0)	163 (61.1)		647 (51.7)	135 (57.7)		494 (40.3)	197 (47.1)	
Poor (1.9–3.0)	26 (2.1)	11 (4.1)		55 (4.4)	20 (8.5)		35 (2.9)	6 (2.9)	
Toothbrushing frequency *n* ^†^ (%)									
No/occasionally	219 (17.9)	51 (19.1)	0.797	147 (11.8)	42 (18.0)	0.033	138 (11.3)	19 (9.2)	0.643
Once per day	741 (60.6)	156 (58.4)		537 (43.0)	96 (41.0)		433 (35.3)	77 (37.4)	
Twice per day	262 (21.4)	60 (22.5)		565 (45.2)	96 (41.0)		655 (53.4)	110 (53.4)	
Subject who performs the toothbrushing *n* ^†^ (%)									
The child, unsupervised	195 (16.3)	65 (25.4)							
The child, supervised by an adult	772 (64.4)	168 (65.6)	<0.001	N/A			N/A		
An adult	231 (19.3)	23 (9.0)							
Frequency of consumption of sugary food (e.g., biscuits, cake, chocolate) *n* ^†^ (%)									
≤Once per day	1048 (88.7)	220 (86.3)							
≥Twice per day	134 (11.3)	35 (13.7)	0.285	Ν/A			Ν/A		
Frequency of consumption of confectionery products (e.g., lollipops, sugary chewing gums, candies) *n* ^†^ (%)									
≤Once per day	1166 (98.0)	240 (93.4)	<0.001	N/A			N/A		
≥Twice per day	23 (2.0)	17 (6.6)							
Frequency of consumption of sugar-sweetened beverages (e.g., packaged juices, chocolate milk, sugary milk *n* ^†^ (%)									
≤Once per day	1142 (96.2)	234 (90.3)	<0.001	N/A			N/A		
≥Twice per day	46 (3.8)	25 (9.7)							

^†^ Number of observations may be less than the total due to missing data. ^‡^ Pearson’s chi-square test. The distribution of the Greek 12- and 15-year-olds according to sociodemographic indicators (gender, location and parental education level), toothbrushing frequency and DI-S scores was adapted from Diamanti et al. (2021) [11,12].

**Table 6:** Effect of ethnic background on the d_3–6_mfs/D_3–6_MFS of the total population of 5-, 12- and the 15-year-old children and adolescents (both of native Greek and of immigrant origin) that were examined (negative binomial regression analyses).

Dependent Variable	Independent Variable								
		5-year-olds							
		Crude (*n* = 1489)				Adjusted (*n* = 1450)			
d_3–6_mfs	Ethnic background (ref. Greek)	B	IRR	95% C.I.	*p*-Value	B	IRR ^†^	95% C.I.	*p*-Value
		0.792	2.21 ^1^	(1.77. 2.76)	<0.001	0.479	1.61 ^1^	(1.22. 2.14)	0.001
		12-year-olds							
		Crude (*n* = 1486)				Adjusted (*n* = 1460)			
D_3–6_MFS	Ethnic background (ref. Greek)	B	IRR	95% C.I.	*p*-Value	B	IRR ^†^	95% C.I.	*p*-Value
		0.494	1.64 ^1^	(1.37. 1.95)	<0.001	0.241	1.27 ^1^	(1.07. 1.51)	0.006
		15-year-olds							
		Crude (*n* = 1434)				Adjusted (*n* = 1422)			
D_3–6_MFS	Ethnic background (ref. Greek)	B	IRR	95% C.I.	*p*-Value	B	IRR ^†^	95% C.I.	*p*-Value
		0.089	1.09 ^1^	(0.90. 1.32)	0.357	0.099	1.10 ^1^	(0.90. 1.35)	0.331

^†^ Model adjusted for gender, location, parental education level, toothbrushing frequency and DI-S score. ^1^ Immigrant. IRR: incidence rate ratio.

## Data Availability

Data supporting reported results are available upon reasonable request to the Scientific Coordinator of the pathfinder survey, Constantine J. Oulis.

## Appendix A

**Table A1:** Distribution of 5-year-old children of immigrant and of native Greek origin according to having a history of nocturnal baby bottle feeding practice.

	Greeks (*n* = 1222)	Immigrants (*n* = 267)	*p*-Value ^‡^
Nocturnal baby bottle feeding practice *n* ^†^ (%)			
No	1069 (90.8)	212 (85.5)	0.029
Yes	108 (9.2)	36 (14.5)	

^†^ Number of observations is less than the total due to missing data. ^‡^ Pearson’s chi-square test.
